# Macrophages inhibit and enhance endometriosis depending on their origin

**DOI:** 10.1073/pnas.2013776118

**Published:** 2021-02-03

**Authors:** Chloe Hogg, Kavita Panir, Priya Dhami, Matthew Rosser, Matthias Mack, Daniel Soong, Jeffrey W. Pollard, Stephen J. Jenkins, Andrew W. Horne, Erin Greaves

**Affiliations:** ^a^Medical Research Council Centre for Reproductive Health, The Queen’s Medical Research Institute, The University of Edinburgh, Edinburgh EH16 4TJ, United Kingdom;; ^b^Centre for Early Life, Warwick Medical School, University of Warwick, CV2 2DX Coventry, United Kingdom;; ^c^Department of Internal Medicine II–Nephrology, University Hospital Regensburg, 93042 Regensburg, Germany;; ^d^Centre for Inflammation Research, The Queen’s Medical Research Institute, The University of Edinburgh, Edinburgh EH16 4TJ, United Kingdom

**Keywords:** lesion, phenotype, ontogeny

## Abstract

Macrophages are intimately involved in the pathophysiology of endometriosis, a chronic inflammatory disorder characterized by the growth of endometrial-like tissue (lesions) outside the uterus. By combining genetic and pharmacological monocyte and macrophage depletion strategies we determined the ontogeny and function of macrophages in a mouse model of induced endometriosis. We demonstrate that lesion-resident macrophages are derived from eutopic endometrial tissue, infiltrating large peritoneal macrophages (LpM) and monocytes. Furthermore, we found endometriosis to trigger continuous recruitment of monocytes and expansion of CCR2+ LpM. Depletion of eutopic endometrial macrophages results in smaller endometriosis lesions, whereas constitutive inhibition of monocyte recruitment significantly reduces peritoneal macrophage populations and increases the number of lesions. Reprogramming the ontogeny of peritoneal macrophages such that embryo-derived LpM are replaced by monocyte-derived LpM decreases the number of lesions that develop. We propose a putative model whereby endometrial macrophages are “proendometriosis” while newly recruited monocyte-derived macrophages, possibly in LpM form, are “antiendometriosis.” These observations highlight the importance of monocyte-derived macrophages in limiting disease progression.

## Significance

Endometriosis is a chronic, incurable inflammatory disorder impacting 190 million women worldwide. Immune cells called macrophages are implicated in promoting endometriosis. Macrophages have different origins and their origin can dictate function. In this study we demonstrate that endometriotic lesion-resident macrophages are derived from the uterine lining (endometrium), the abdominal (peritoneal) cavity, and recruited bone-marrow precursors (monocytes). Endometriosis triggers continuous recruitment of monocytes that differentiate into macrophages that differ from those usually present within the peritoneal cavity. By depleting different populations, we demonstrate that endometrial macrophages are “proendometriosis” while monocyte-derived peritoneal macrophages are “antiendometriosis” acting to protect the cavity from lesion establishment. In the future, immune-based therapies may allow targeting of prodisease macrophages and/or harnessing of antiendometriosis macrophages in endometriosis.

Macrophages are exceptionally diverse cells present in all tissues of the body that perform functions vital for immunity, development, tissue homeostasis, and repair following injury. They modify their role depending on signals received from their local microenvironment and accordingly exhibit high degrees of transcriptional and phenotypic heterogeneity and tissue-specific function (1, 2). Macrophages differ in their ontogeny. While early studies suggested macrophages were continually replaced by circulating blood monocytes, more recent lineage-tracing experiments demonstrated that most tissue-resident macrophages (exceptions include gut, dermis, and heart) are derived from embryonic precursors that seed tissues prior to birth and are maintained by self-renewal or longevity (3–5). Tissue-resident and monocyte-derived macrophages play distinct roles both in health and disease (6). Usually, tissue-resident macrophages play tissue-specific homeostatic roles as well as core functions such as clearance of dying cells. On the other hand, monocyte-derived macrophages that are recruited to tissues during inflammation secrete proinflammatory cytokines, help clear infection, and regulate the immune response (6). Thus, in pathological situations, macrophages are a heterogenous population. For example, during acute liver injury hepatic resident macrophages (Kupffer cells) become activated and recruit monocytes that initially promote liver injury but then subsequently differentiate into inflammatory macrophages and help resolve injury and drive regeneration (7). In pancreatic cancer, both tissue-resident and monocyte-derived macrophages populate the tumor and increase in density as the cancer progresses. The two populations are transcriptionally diverse and depletion studies revealed that only tissue-resident macrophages are responsible for driving tumor progression (8). These findings also highlight that under disease-modified conditions tissue-resident macrophages can become adapted such that they promote disease. Conversely, in other cancers (e.g., breast), monocytes are recruited to the tumor and differentiate into tumor-associated macrophages that drive disease (1).

The peritoneal cavity hosts two main macrophage populations: a predominant population expressing high levels of epidermal growth factor-like module-containing mucin-like hormone receptor-like 1 (EMR1/F4/80) and low levels of major histocompatibility class II (MHCII) known as large peritoneal macrophages (LpM), and a less abundant population that are F4/80^lo^, MHCII^hi^ [small peritoneal macrophages (SpM) (9)]. LpM are considered to be tissue-resident macrophages and are largely embryonically derived; however, it is now understood that they are gradually replaced by monocytes over time in a sexually dimorphic manner that occurs more quickly in males (10, 11). The transcription factor GATA6 is highly expressed by all LpM in response to retinoic acid and drives a significant proportion of the tissue-specific transcriptional signature of these cells (12). The SpM population is a more heterogeneous population comprising monocyte-derived macrophages and dendritic cells that are continually replenished from the blood (10, 13). Inflammatory challenge in the peritoneal cavity can result in recruitment of large numbers of inflammatory macrophages that are transcriptionally distinct from SpM (14), and a loss in LpM (the so-called macrophage disappearance reaction), which results from formation of cell aggregates (15) or cell death (16). An exception to this is helminth infections which are characterized by expansion of LpM in response to local Th2 cytokine production (14, 17–20).

Endometriosis is a chronic inflammatory condition where tissue similar to the endometrium grows ectopically, usually in the peritoneal cavity as “lesions” (21). The condition impacts an estimated 190 million women worldwide and is associated with debilitating pelvic pain and infertility (22). Currently, therapeutic options are very limited, with the gold-standard treatments being surgical removal of lesions or suppression of ovarian hormones. Surgery is associated with high recurrence rates and ovarian suppression is contraceptive and often has unwanted side effects. A high abundance of macrophages is reported both in the peritoneal cavity and in lesions of women with endometriosis (23). It is clear that macrophages are intrinsically linked with the pathophysiology of endometriosis, where they enhance establishment, proliferation, and vascularisation of lesions (24, 25). They are also critical in promoting innervation of lesions and concomitant sensitization of nerve fibers, thus contributing to pain in the condition (26, 27). Evidence from a syngeneic mouse model of induced endometriosis indicates that donor endometrial macrophages as well as host-derived macrophages can be identified in endometriosis lesions (28). However, the exact origins of the host-derived macrophages and specific functions of the different populations remain to be determined.

Since macrophages play such a key role in many aspects of the pathophysiology of endometriosis they represent an attractive therapeutic target. However, the development of a viable immune-therapy targeting “disease-promoting” macrophages or enhancing the function of “protective” macrophages requires a comprehensive understanding of the origin and function of lesion-resident and associated peritoneal macrophages. In this study we have characterized the origin of endometriosis lesion-resident macrophages and examined the dynamics of peritoneal cavity macrophage populations. Finally, we have used a combination of transgenic and pharmacological approaches to selectively deplete different populations of macrophages to assess their impact on development of endometriosis lesions.

## Results

### Endometriosis Lesion-Resident Macrophages Have Multiple Origins.

**Fig. 1.**
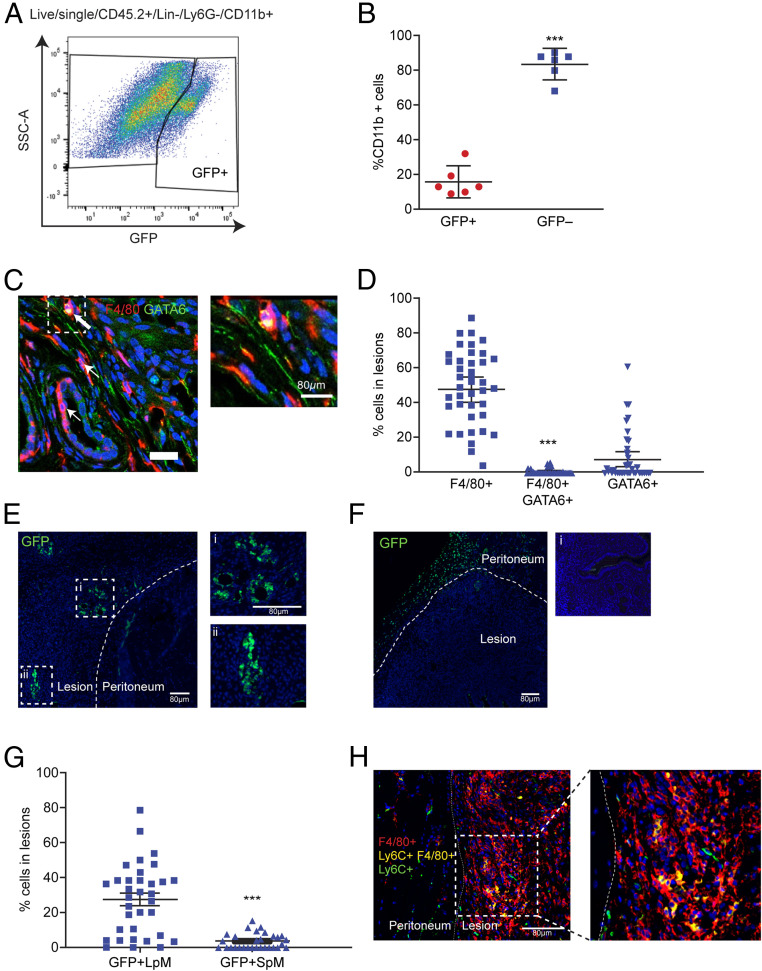
Lesion-resident macrophages have different origins. Donor endometrial tissue from MacGreen mice was injected into the peritoneal cavity of wild-type recipient mice to assess incorporation of endometrial macrophages into lesions. Lesions were collected at 2 wk after tissue injection in each of the separate studies presented in this figure. (*A*) Expression of GFP by lesion-resident macrophages recovered from MacGreen (donor) to wild-type (recipient) endometrial transfers (*n* = 6). (*B*) Quantification of donor endometrial-derived (GFP+) macrophages vs. recipient-derived (GFP−) macrophages. Dual immunodetection for identification of LpM in lesions. (*C*) Dual immunodetection for F4/80 (red) and GATA6 (green; *n* = 7 mice [10 lesions]). Thick arrows indicate dual-positive cells and thin arrows indicate GATA6− macrophages. (*D*) Quantification of F4/80+, dual-positive and GATA6+ cells in lesions. Fewer than 1% of cells were dual-positive for F4/80 and GATA6. Adoptive transfers of MacGreen peritoneal macrophages into wild-type mice with GFP immunodetection to assess incorporation of LpM and SpM into lesions. (*E* and *F*) Immunofluorescence for GFP on lesions collected following adoptive transfer of approximately 1 × 10^6^ LpM (*E*) or SpM (*F*) isolated from MacGreen mice. Curved dotted line indicates the boundary between peritoneal and lesion tissue. In *E*, i and ii show magnified images; in *F*, i shows a negative control. (*G*) Quantification of GFP+ LpM and SpM in lesions. Dual immunodetection for Ly6C+ monocytes in lesions. (*H*) Dual immunodetection for F4/80 (red) and Ly6C (green) performed on mouse lesions. Data are presented as mean with 95% confidence intervals. Statistical significance was determined using a Student’s *t* test. ***P < 0.001.

We induced endometriosis in wild-type C57BL/6 mice by injecting “menses-like” endometrium from MacGreen donor mice (*Csf1r-eGFP*; macrophages are green fluorescent protein–positive [GFP+]) (29) into the peritoneal cavity as previously described (28). In MacGreen mice all monocytes and macrophages in the shed menses-like endometrium are GFP+ (30). Two weeks following tissue injection, endometriosis lesions were collected, digested, and analyzed by flow cytometry. GFP+ macrophages could be detected among cluster of differentiation (CD)45+, lineage− (CD3, CD19, CD335, sialic acid-binding immunoglobulin-type lectin F [SIGLEC-F]), Lymphocyte antigen 6 complex, locus G6D (Ly6G)−, integrin alpha M (CD11b)+ lesion cells (Fig. 1*A*). These data indicate that macrophages derived from the donor endometrium reside within lesions and verifies our previous findings using immunodetection (28). Endometrial-derived macrophages (GFP+) represented 16.0% (SD ± 8.6) of lesion-resident macrophages, while the remaining 84.0% (GFP−; SD ± 8.4) were host-derived infiltrating populations (Fig. 1*B*). Next, we sought to determine the origin of the host-derived infiltrating cells. We investigated infiltration of LpM into endometriosis lesions using dual immunodetection for F4/80 (red) and the transcription factor GATA binding protein 6 (GATA6; LpM marker, green; Fig. 1*C*). Quantification of dual-positive cells revealed that less than 1.0% of lesion-resident cells were derived from LpM (Fig. 1*D*). To verify these findings, we performed adoptive transfer of GFP+ LpM isolated by fluorescent activated cell sorting (FACS) from MacGreen mice (LpM and SpM were determined based on expression of F4/80 and MHCII, discussed below). GFP+ macrophages could be easily detected in lesions (Fig. 1*E*), suggesting that LpM infiltrate endometriosis lesions and lose expression of GATA6, consistent with a change in phenotype within the lesion microenvironment. Conversely, very few GFP+ macrophages were detected in lesions following adoptive transfer of SpM isolated from MacGreen mice, suggesting that significant trafficking of peritoneal macrophages to lesions was restricted to the LpM population (Fig. 1*F*). Interestingly, GFP+ SpM were instead observed located to the peritoneum adjacent to lesions. Quantification of GFP immunofluorescence revealed that a mean of 27.4% of cells in lesions were derived from LpM and a mean of 3.6% were derived from SpM (Fig. 1*G*). To assess infiltration of monocytes into lesions we used dual immunodetection for F4/80 (red) and Lymphocyte antigen 6 complex, locus C (Ly6C; green). We identified Ly6C-positive lesion-resident monocytes and a population of dual-positive cells (yellow) were also detected, indicating that monocytes infiltrated lesions and differentiated in situ into macrophages (Fig. 1*H*).

### Continuous Recruitment and Contribution of Monocytes to Peritoneal Macrophage Populations in Mice with Induced Endometriosis.

**Fig. 2.**
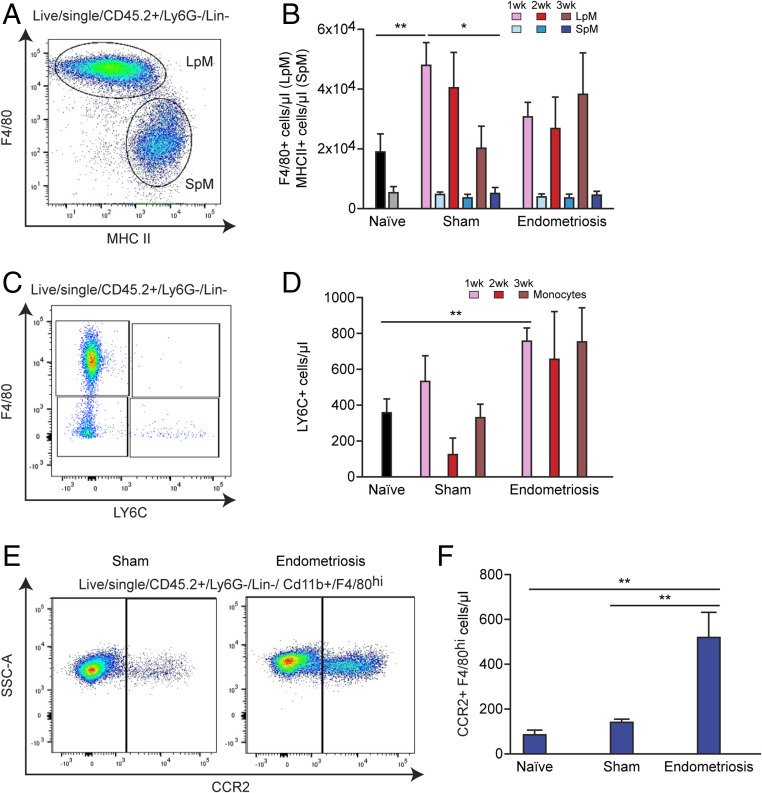
Monocyte recruitment and replenishment of peritoneal macrophage pools in mice with induced endometriosis. (*A*) LpM (F4/80^hi^, MHCII^lo^) and SpM (F4/80^lo^, MHCII^hi^) populations in the peritoneal fluid of mice. (*B*) Quantification of LpM and SpM of sham (*n* = 6 to 8 at each time point) and endometriosis mice at 1 (*n* = 6), 2 (*n* = 8), and 3 wk (*n* = 16) after endometrial tissue injection compared to naïve mice (*n* = 12). (*C*) Flow plot indicating gating of monocytes (F4/80^lo^, Ly6C^hi^) in peritoneal lavage fluid. (*D*) Quantification of monocyte numbers of sham and endometriosis mice at 1, 2, and 3 wk after tissue injection. (*E*) Flow plot demonstrating expression of Ccr2 on F4/80^hi^ macrophages in peritoneal lavage fluid from sham vs. endometriosis mice (2 wk after tissue injection). (*F*) Quantification of CCR2+, F4/80^hi^ cells from endometriosis mice (*n* = 5) compared to sham (*n* = 4) and naïve (*n* = 4) mice. Data are presented as mean ± SEM. Statistics were determined using a one-way ANOVA and a Tukey post hoc test. *P < 0.05, **P < 0.01.

Next, we investigated macrophage populations present in the peritoneal cavity of mice with induced endometriosis. From CD45+, Ly6G−, lineage− cells, LpM and SpM were determined based on expression of F4/80 and MHCII (Fig. 2*A*). At 1 wk after tissue injection, LpM (F4/80^hi^, MHCII^lo^) numbers were significantly higher in sham mice (ovariectomized and treated with estradiol valerate and subject to intraperitoneal [i.p.] injection of phosphate-buffered saline instead of endometrial tissue) compared to naïve mice (mean ± SEM = 48,399 ± 7,361 cells per microliter in sham and 18,969 ± 5,989 cells per microliter in naïve; Fig. 2*B*; *P* < 0.01). At 3 wk, LpM numbers in sham animals had decreased compared to 1 wk (*P* < 0.05). In contrast, no significant differences were found in the LpM population in mice with endometriosis compared to naïve and sham animals or within the endometriosis group at different time points. However, a trend can be observed indicating a moderate decrease in LpM numbers at 1 wk after tissue injection in endometriosis mice compared to sham. This suggests that, compared to sham controls, there may be some loss of LpM following transfer of endometrial tissue, and this could be attributed to LpM trafficking into lesions or possible clotting of LpM as seen in peritonitis models (31). By 3 wk the trend toward decreased LpM in endometriosis mice compared to sham is no longer evident and is consistent with our previous reports demonstrating an increase in LpM at 3 wk in mice with endometriosis (26). SpM (F4/80^lo^, MHCII^hi^) numbers were consistent between all groups of animals and time points. Ovariectomy alone can have striking impacts on macrophage pools present in the peritoneal cavity and leads to increased macrophage replenishment (11). We repeated our results using intact recipient mice and confirmed that in this minimally invasive model there was no distinct shift in the ratio of SpM to LpM, suggesting that transfer of endometrial tissue does not disrupt the normal balance of peritoneal macrophages (*SI Appendix*, Fig. S1*A*). Monocytes in the peritoneal cavity were identified by expression of Ly6C (detection of classical monocytes; Fig. 2*C*) and numbers were significantly increased in mice with induced endometriosis at 1 wk after tissue injection compared to naïve mice (Fig. 2*D*; *P* < 0.01). Monocyte numbers remained elevated between weeks 1 and 3 in mice with induced endometriosis, suggesting continuing recruitment to the peritoneal cavity as a consequence of the presence of endometriotic lesions. To begin to investigate the fate of monocytes recruited to the peritoneal cavity of mice with induced endometriosis, we evaluated expression of C-C chemokine receptor type 2 (CCR2; mediates monocyte chemotaxis/recruitment of monocytes) by F4/80^hi^ (LpM) macrophages in the peritoneal cavity using flow cytometry. Significantly elevated numbers of F4/80+, CCR2+ macrophages were recorded in mice with induced endometriosis (Fig. 2 *E* and *F*), suggesting that increased numbers of monocyte-derived LpM are evident in mice with endometriosis. In steady-state conditions, long-lived embryo-derived peritoneal macrophages express T cell immunoglobulin and mucin domain containing 4 (TIM4), while recently monocyte-derived LpM do not (10); thus, to further validate our hypothesis we ascertained TIM4 expression in F4/80^hi^ LpM in intact mice. We found a significant reduction in TIM4^hi^ LpM at weeks 2 and 3 compared to week 1 (*SI Appendix*, Fig. S1*B*; *P* < 0.05) and a concomitant increase in TIM4^lo^ LpM at weeks 2 (*P* < 0.05) and 3 (*P* < 0.01) compared to week 1 (*SI Appendix*, Fig. S1*B*). This finding is consistent with heightened monocyte input into the LpM pool in endometriosis. We also observed a significant reduction in the proportion of F4/80^lo^, MHCII− cells within the CD11b+ peritoneal population between week 1 and week 3 after endometrial tissue injection (*SI Appendix*, Fig.S1 *C* and *D*). Only a minor proportion of these cells express Ly6C (less than 2% of CD11b cells), indicating that this population may be a transitory state between monocyte and LpM.

### Endometrial Macrophage Depletion Leads to Reduced Endometriotic Lesion Size.

**Fig. 3.**
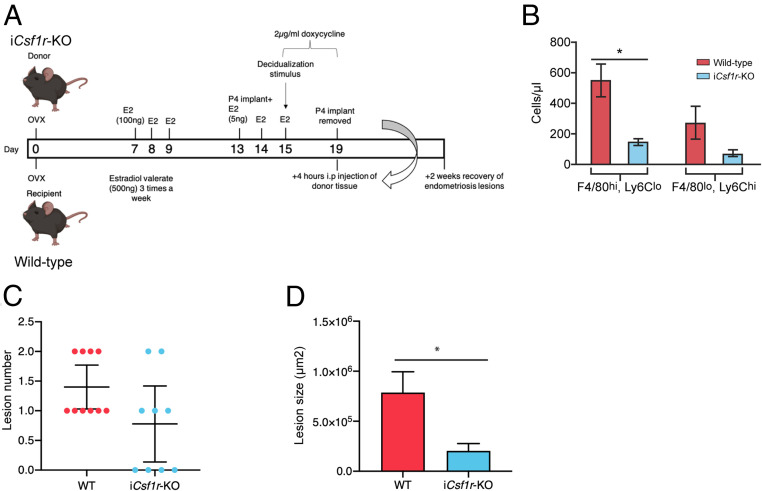
Endometrial macrophage depletion impacts lesion size. (*A*) Schematic demonstrating timing of doxycycline administration to i*Csf1r*-KO donor mice. Donor endometrium was generated in i*Csf1r*-KO mice, with doxycycline administered to donor mice from days 15 to 19 to deplete endometrial macrophages prior to recovery of endometrium and i.p. transfer to wild-type recipients. Lesions were recovered 2 wk after tissue injection. (*B*) Quantification of F4/80^hi^, Ly6C^lo^ macrophages and Ly6C^hi^, F4/80^lo^ monocytes in donor endometrium from wild-type and iCsf1r-KO mice. (*C*) Number of lesions recovered from wild-type recipient mice receiving either wild-type (*n* = 10) or i*Csf1r*-KO endometrium (*n* = 9) (from two independent experiments). (*D*) Area of lesions recovered from mice receiving either wild-type or i*Csf1r*-KO endometrium. Data are presented as mean ± SEM or 95% confidence intervals (*C*). Statistical significance was determined using a Student’s *t* test. *P < 0.05.

To dissect the role of macrophages with different ontogenies in endometriosis we used a number of depletion strategies. To deplete endometrial macrophages, doxycycline was administered to i*Csf1r*-KO donor mice from days 15 to 19 after ovariectomy (Fig. 3*A*), such that we could achieve macrophage depletion by inducibly ablating expression of *Csf1r* (32). Pretransfer analysis revealed that the endometrium was significantly depleted of F4/80^hi^ macrophages (Fig. 3*B*; *P* < 0.05). Following transfer of macrophage-depleted donor endometrial tissue to wild-type recipients, there was no difference in the number of lesions recovered after 2 wk (Fig. 3*C*); however, the lesions recovered were significantly smaller than those from mice receiving wild-type endometrium (Fig. 3*D*; *P* < 0.05). These data suggest that macrophages within endometrial tissue promote the growth of endometriosis lesions but do not significantly impact the attachment of endometrial tissue to the peritoneal lining.

### “Monocytopenic” Mice with Induced Endometriosis Had an Increased Number of Lesions.

**Fig. 4.**
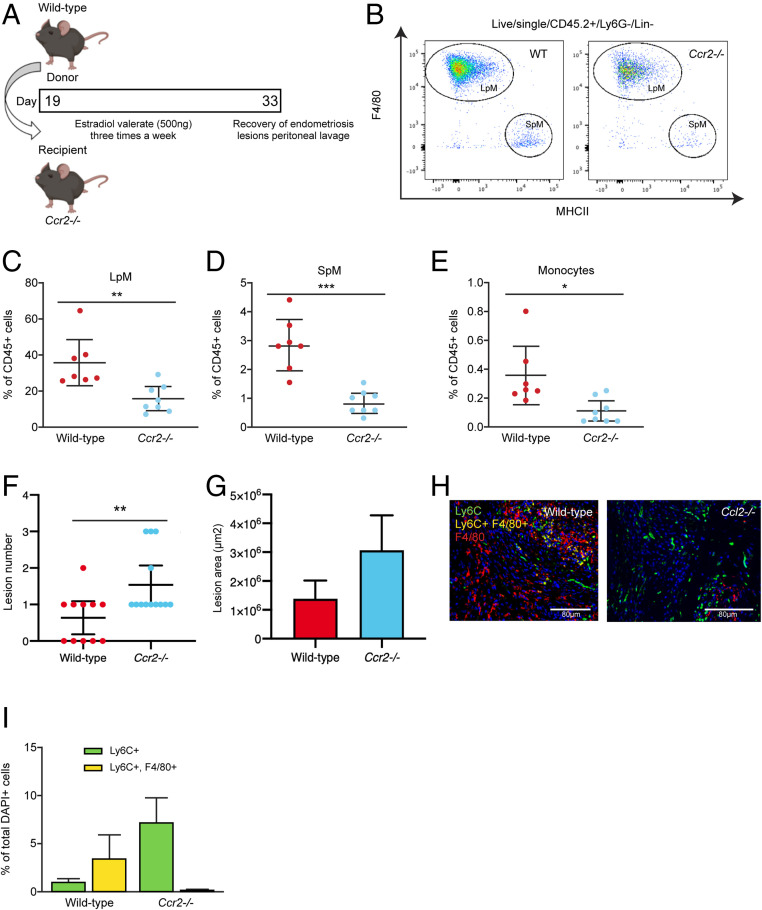
”Monocytopenic” mice with induced endometriosis establish more lesions. (*A*) Schematic demonstrating experimental design. Wild-type donor endometrium was generated as previously shown (Fig. 3*A*) and injected i.p. into ovariectomized *Ccr2*^−/−^ recipients. Wild-type recipients were also used as controls. Lesions were recovered 2 wk after tissue injection. (*B*) Flow plot indicating gating and number of LpM (F4/80^hi^) and SpM (MHCII^hi^) in peritoneal lavage fluid recovered from wild-type (*n* = 7) and *Ccr2*^−/−^ mice (*n* = 8) with induced endometriosis. (*C*–*E*) Quantification of (*C*) LpM, (*D*) SpM, and (*E*) monocytes (Ly6C^hi^) in peritoneal lavage fluid. (*F*) Number of lesions recovered from wild-type (*n* = 11) and *Ccr2*^−/−^ (*n* = 13) mice with induced endometriosis (from three independent experiments). (*G*) Size of lesions recovered from wild-type and *Ccr2*^−/−^ mice with induced endometriosis. (*H*) Dual immunodetection for Ly6C (green) and F4/80 (red) on lesions recovered from wild-type and Ccr2^−/−^ mice. (*I*) Quantification of monocytes (Ly6C+; yellow bars) and monocyte-derived macrophages (Ly6C+, F4/80+; green bars) in lesions recovered from wild-type and *Ccr2*^−/−^ mice. Data are presented as mean ± SEM or 95% confidence intervals (*F*). Statistical significance was determined using a Student’s *t* test or Mann–Whitney *U* test. *P < 0.05, **P < 0.01, ***P < 0.001.

**Fig. 5.**
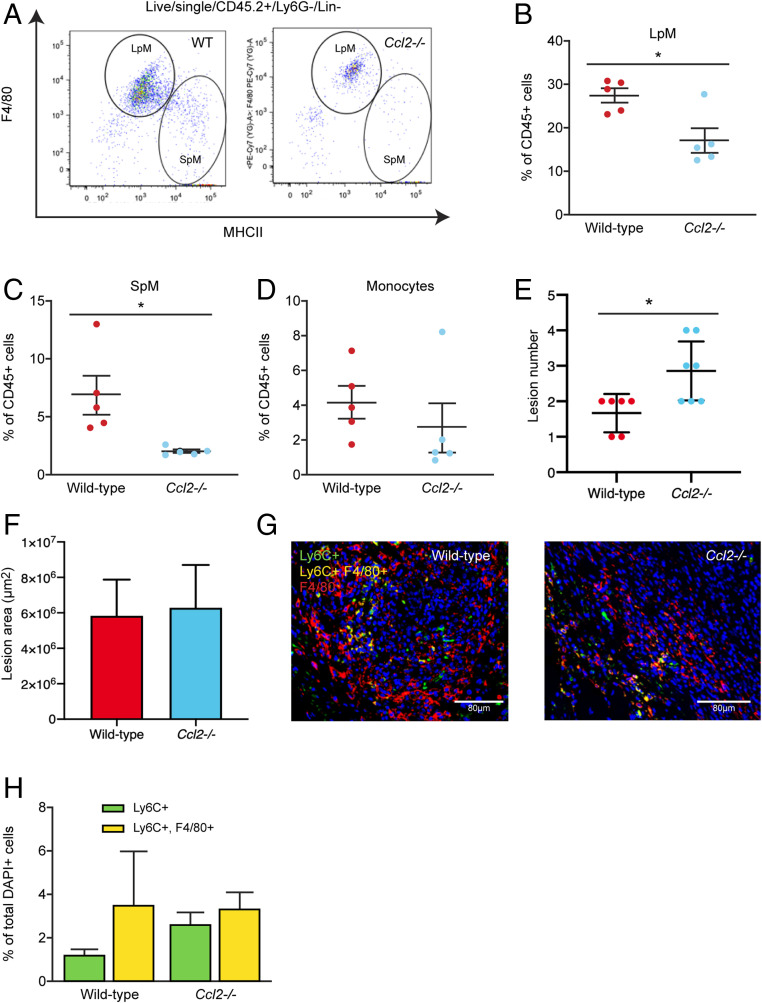
More lesions are evident in *Ccl2*^−/−^ mice. Wild-type donor endometrium was generated as previously shown (Fig. 3*A*) and injected i.p. into ovariectomized *Ccl2*^−/−^ recipients as in the previous figure. Wild-type recipients were also used as controls. Lesions were recovered 2 wk after tissue injection. (*A*) LpM and SpM populations in peritoneal lavage fluid from wild-type and *Ccl2*^−/−^ mice with induced endometriosis. (*B*–*D*) Quantification of (*B*) LpM, (*C*) SpM, and (*D*) monocytes in peritoneal lavage fluid from wild-type (*n* = 5) and *Ccl2*^−/−^ (*n* = 5) mice with induced endometriosis. (*E*) Number of lesions recovered from wild-type and *Ccl2*^−/−^ mice. (*F*) Size of lesions recovered from wild-type (*n* = 6) and *Ccl2*/- (*n* = 7) mice with induced endometriosis (from two independent experiments). (*G*) Dual immunodetection for Ly6C (green) and F4/80 (red) on lesions recovered from wild-type and *Ccl2*^−/−^ mice. (*H*) Quantification of monocytes (Ly6C+) and monocyte-derived macrophages (Ly6C+, F4/80+) in lesions recovered from wild-type and *Ccl2*^−/−^ mice. Data are presented as mean ± SEM or 95% confidence intervals (*E*). Statistical significance was determined using a Student’s *t* test or Mann–Whitney *U* test. *P < 0.05.

Previous studies have inferred a role for recipient peritoneal macrophages in lesion development since continual i.p. delivery of clodronate liposomes or anti-F4/80 antibody throughout the growth phase resulted in smaller lesions (24). Further, i.p. transfer of bone marrow (BM)-derived macrophages could enhance or inhibit growth dependent on polarization of macrophages in vitro prior to transfer (24). However, in addition to embryo-derived resident peritoneal macrophages, it is possible these methods also deplete endometrial macrophages (in transferred endometrial tissue) and recruited monocyte-derived cells. Notably, these studies showed that liposome-mediated depletion of embryonic resident peritoneal macrophages prior to endometrial transplantation did not significantly affect lesion development (24), suggesting the role of embryonic LpM is, at best, redundant. Hence, we next aimed to prevent recruitment of host monocytes to the peritoneal cavity and lesions using *Ccr2* null monocytopenic recipient mice. LpM, SpM, and monocytes were all significantly reduced in the peritoneal lavage fluid of *Ccr2*^*−/−*^ mice with induced endometriosis compared to wild-type mice with endometriosis (a reduction of 56.4%, 70.5%, and 69.0% in mean values respectively; Fig. 4 *A*–*E* and *SI Appendix*, Fig. S2 *A*–*C*). This supports the concept that in our mouse model of endometriosis monocytes are continually recruited and contribute to the LpM and SpM pools. Furthermore, while lesion size was not different between the two groups (Fig. 4*G*), *Ccr2*^−/−^ mice had significantly more lesions (*P* < 0.01) compared to wild-type mice (Fig. 4*F*). This indicates that monocytes or monocyte-derived macrophages normally protect against establishment of lesions. To validate these findings, we repeated the experiment in Chemokine (C-C motif) ligand 2 null (*Ccl2*^−/−^) mice. In *Ccl2*^−/−^ mice with endometriosis, LpM and SpM were significantly depleted compared to wild-type mice with endometriosis (a reduction of 37.9% and 69.8% in mean values respectively; Fig. 5 *A*–*C*; *P* < 0.05 and *SI Appendix*, Fig. S2 *D*–*F*). We observed a concomitant increase in the number of lesions recovered from *Ccl2*^−/−^ mice (Fig. 5*E*; *P* < 0.05). Strikingly, *Ccr2*^−/−^and *Ccl2*^−/−^ mice with induced endometriosis were still able to recruit Ly6C+ monocytes from the BM to the peritoneal cavity despite monocytes being absent in the peritoneal lavage of naïve and sham monocytopenic mice (*SI Appendix*, Fig. S3). Moreover, in lesions recovered from *Ccr2*^−/−^ and *Ccl2*^−/−^ mice we could still detect monocytes using immunodetection (Figs. 4 *H* and *I* and 5 *G* and *H*), indicating that there is redundancy in the CCL2–CCR2 axis in the presence of endometriosis lesions and suggesting that monocytes may also be recruited via another mechanism.

### Transient Depletion of Monocytes Did Not Impact Establishment of Endometriotic Lesions.

**Fig. 6.**
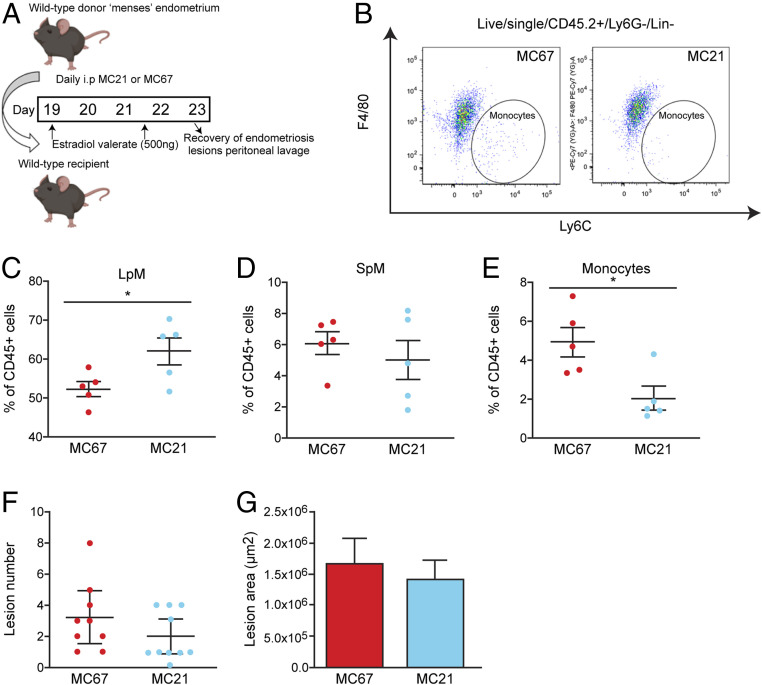
A function-blocking Ccr2 mAb reduces monocyte numbers without significant impact on lesion number. (*A*) Schematic showing the experimental design; mice with induced endometriosis were treated with a control IgG (MC67; *n* = 9) or a function-blocking CCR2 mAb (MC21; *n* = 10, from two independent experiments) 6 h prior to endometrial tissue injection and daily for an additional 4 d. Lesions were recovered 5 d after tissue injection. (*B*) Flow plot demonstrating the numbers of Ly6C^hi^ monocytes in mice with induced endometriosis treated with MC67 or MC21. (*C*–*E*) Quantification of (*C*) LpM, (*D*) SpM, and (*E*) monocytes in the peritoneal lavage fluid of mice with induced endometriosis. (*F*) Number of lesions recovered from mice treated with MC67 or MC21. (*G*) Size of lesions recovered from mice treated with MC67 or MC21. Data are presented as mean ± SEM or 95% confidence intervals (*F*). Statistical significance was determined using a Student’s *t* test or a Mann–Whitney *U* test. *P < 0.05.

Next, we sought to transiently deplete monocytes to ascertain the role of undifferentiated monocytes in lesion development while leaving the peritoneal macrophage populations relatively unaltered. We depleted monocytes using a function-blocking CCR2 monoclonal antibody (MC21) injected into the peritoneal cavity of mice with induced endometriosis. Mice were injected with MC21 6 h prior to transfer of endometrial tissue and every following day for 4 d (Fig. 6*A*). Compared to a control immunoglobulin G (IgG) antibody (MC67), MC21 triggered an expansion of LpM (Fig. 6*C*; *P* < 0.05), did not modify SpM (Fig. 6*D*), and significantly reduced the number of monocytes in the peritoneal cavity (Fig. 6 *B* and *E*; *P* < 0.05). In mice with induced endometriosis treated with MC21 there was no difference in lesion number or size compared to mice treated with control MC67 (Fig. 6 *F* and *G*). These results rule out the possible contribution of recently recruited monocytes to the “antiendometriosis” function of monocyte-derived cells observed in Figs. 4 and 5.

### Reprogramming the Ontogeny of Macrophages in the Peritoneal Cavity of Mice with Induced Endometriosis Leads to Reduced Lesion Size.

**Fig. 7.**
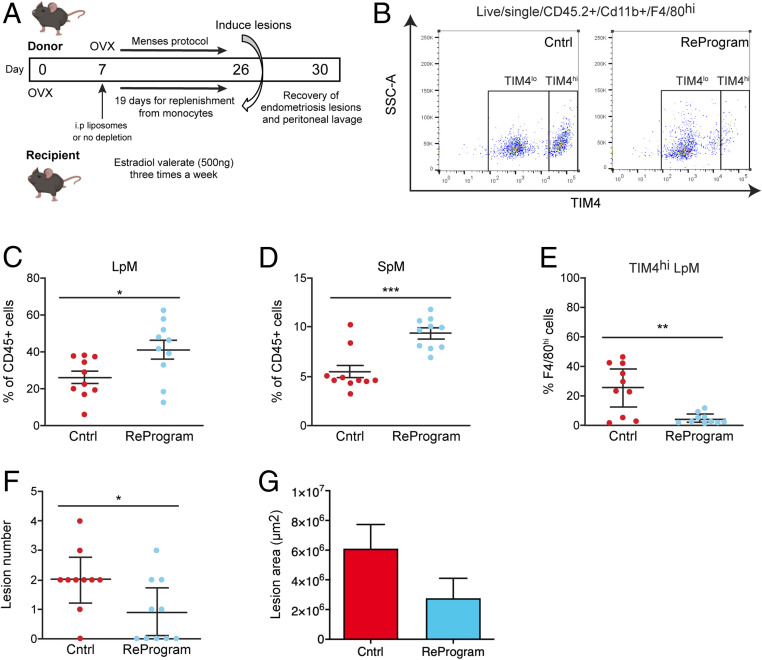
Reprogramming the ontogeny of peritoneal macrophages leads to establishment of fewer lesions. (*A*) Schematic showing the experimental design; 7 d after ovariectomy experimental mice were administered i.p. with liposomal clodronate to deplete all peritoneal macrophages (*n* = 10). Nineteen days were allowed for replenishment of the niche prior to transfer of endometrial tissue on day 26. Lesions were recovered 5 d after tissue injection. Control mice (*n* = 10) did not receive liposomal clodronate (single independent experiment). (*B*) Flow plot demonstrating the numbers of TIM4^hi^ and TIM4^lo^ LpM in control mice with induced endometriosis or mice with reprogrammed cavities with induced endometriosis. (*C*–*E*) Quantification of (*C*) LpM, (*D*) SpM, and (*E*) TIM4^hi^ LpM in the peritoneal lavage fluid of mice with induced endometriosis. (*F*) Number of lesions recovered from control mice and those with reprogrammed cavities. (*G*) Size of lesions recovered from control mice and those with reprogrammed cavities. Data are presented as mean ± SEM or 95% confidence intervals (*F*). Statistical significance was determined using a Student’s *t* test or a Mann–Whitney *U* test. *P < 0.05, **P < 0.01, ***P < 0.001.

Next, we aimed to confirm the protective role of monocyte-derived LpM against establishment of lesions by reprogramming the ontogeny of macrophages in the peritoneal cavity. Seven days following ovariectomy we depleted peritoneal macrophages using liposomal clodronate and allowed 19 d (33) for replenishment of the niche from monocytes prior to injection of donor endometrial tissue (Fig. 7*A*). Compared to control mice with induced endometriosis, those with reprogrammed cavities exhibited elevated LpM and SpM (Fig. 7 *C* and *D*; *P* < 0.05 and *P* < 0.001 respectively). Moreover, endometriosis mice with reprogrammed cavities were significantly depleted of TIM4^hi^ LpM (a reduction of 82.7% in mean values compared to control mice with endometriosis; Fig. 7*E*; *P* < 0.01) consistent with replacement of embryo-derived LpM with monocyte-derived LpM (10, 33). In mice with reprogrammed cavities, the number of lesions that developed was significantly reduced, with five mice developing no lesions at all (*P* < 0.05; Fig. 7*F*). There was no difference in lesion area in mice with reprogrammed cavities compared to control mice (Fig. 7*G*). Collectively, these data strongly support the concept that monocyte-derived LpM act to protect the peritoneal cavity when challenged with ectopic endometrial tissue.

## Discussion

The pathophysiology of endometriosis remains enigmatic (21). Although immune cell dysfunction is intrinsically linked with the disorder, our understanding of macrophage origins and respective function remains limited compared with other diseases, such as cancer (34). As the ontogeny of macrophages in diseased tissue is a key determinant of how they respond and contribute to pathogenesis, it is necessary to understand how macrophages derived from different sources impact lesion development. In the current study we have determined, in a mouse model of induced endometriosis, that lesion-resident macrophages are derived from the eutopic endometrium, infiltrating LpM and monocytes. We have demonstrated that endometriosis triggers continuous recruitment of monocytes to the peritoneal cavity and heightened monocyte input into the LpM pool. We show that endometrial macrophages promote the growth but not the establishment of endometriotic lesions, whereas monocyte-derived LpMs play a protective role against lesion development.

The contribution of embryonic-derived and monocyte-derived macrophages to the “tissue-resident” population is different in each tissue (3–5). We previously demonstrated that macrophages present in the endometrium can be detected in lesions recovered from our model of induced endometriosis (28). However, little is known regarding the ontogeny of macrophages in the endometrium. In a mouse model of menstruation, utilizing MacGreen (*Csf1r-EGFP*) mice, Cousins et al. (30) demonstrated that three populations could be distinguished in the endometrium using dual staining for F4/80 and GFP: 1) a population of GFP+, F4/80− cells likely to be infiltrating monocytes, 2) a population of GFP+, F4/80+ cells suggestive of monocyte-derived macrophages, and 3) a population of putative (but not confirmed with lineage tracing) “tissue-resident” macrophages that are GFP−, F4/80+. The cells were localized to areas of breakdown, repair, and remodeling, respectively. The “menses-like” endometrium that we recover from donor mice for transfer into recipient mice is collected at the initiation of the “breakdown” phase and is most likely to consist of monocytes and monocyte-derived macrophages. Moreover, the tissue collected is the “decidual” mass only and does not include the compartment of the uterus where putative “tissue-resident” macrophages are located. Using an inducible *Csf1r* knockout to generate donor endometrium we achieved depletion of macrophages (F4/80^hi^, Ly6C^lo^) and limited the number of monocytes (F4/80^lo^, Ly6C^hi^) in the tissue transferred to recipient mice. We did not observe any difference in the number of lesions formed between recipient mice that received wild-type or macrophage-depleted endometrium; however, we did find that the lesions recovered were significantly smaller in mice receiving macrophage-depleted endometrium, indicating that endometrial macrophages play a critical role in growth of lesions. The breakdown phase of the (donor) endometrium is analogous to the initial inflammatory phase of the wound-healing process where proinflammatory macrophages play a vital role prior in wound repair (35). If we consider endometriotic lesions as chronic wounds that do not fully resolve their inflammation, we may presume that the initial inflammatory phase begins as the endometrium breaks down during menstruation (or within the established lesion during cyclical remodeling) and the repair of the translocated endometrium occurs in the peritoneal cavity, resulting in the formation of lesions. The subsequent phase of the tissue repair process is the proliferative phase: In mouse models of skin injury, macrophage depletion during this phase resulted in granulation tissue that had very few blood vessels and proliferative cells and a significant reduction in wound closure. This indicates that macrophages support this phase by promoting endothelial cell survival and vascularization, which facilitates proliferation (36). We suggest that a similar process occurs in mice receiving endometrium depleted of monocytes and macrophages and this may explain reduced lesion size in recipient mice. Further, previous studies have demonstrated that macrophages do not regulate survival or proliferation of uterine epithelial, stromal, or vascular endothelial cells during the estrus cycle or following exogenous supplementation with estradiol and progesterone (37). This evidence supports the concept that macrophages are implicated in tissue repair after hormone withdrawal or injury (e.g., following endometrial shedding and in endometriosis), as opposed to having a trophic role under steady-state conditions.

The ontogeny of peritoneal macrophages is better characterized. LpM are embryo-derived and long-lived and undergo self-renewal; however, monocytes do continually enter the peritoneal cavity via CCR2, where they continually replenish the SpM compartment and infrequently differentiate into LpM (10). Such replenishment of LpM occurs in a sexually dimorphic manner, occurring more quickly in male compared to female mice who retain their embryo-derived LpM for much longer (10, 16), and it is likely that in adult female mice of the age used in our studies (10 to 12 wk) only 10 to 30% of peritoneal LpM would be derived from adult monocytes (11, 16). In this study we used ovariectomy and estradiol supplementation of recipient animals to allow optimal lesion development. Peritoneal surgery increases the contribution of monocytes to the peritoneal LpM; however, the embryonic component would still be expected to comprise ∼50% of the population at the time of injection of endometrial material. Although monocyte-derived LpM mostly phenocopy embryo-derived LpM, transcriptomically they exhibit some differences. For example, embryo-derived LpM express *Timd4*, while in steady state many monocyte-derived LpM do not, or take significant time to do so (10, 11). Notably, the number of monocytes and CCR2+ LpM was significantly elevated in the peritoneal cavity of mice with induced endometriosis, above levels seen in ovariectomized controls, suggesting monocytes are continually recruited and contribute to LpM pools during lesion development.

**Fig. 8.**
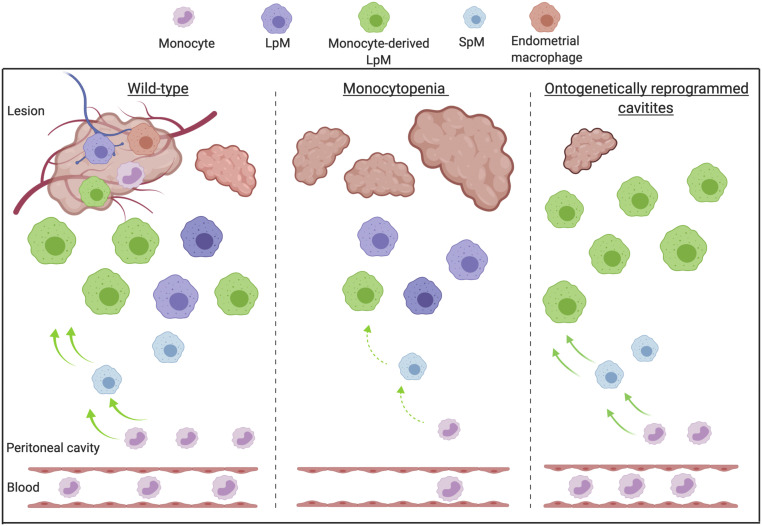
Monocyte-derived macrophages are guardians of the peritoneal cavity in mice with induced endometriosis. Created with https://biorender.com/. Lesion-resident macrophages are a heterogenous population constituted by macrophages that have different origins; endometrial, peritoneal (LpM), and recruited monocytes that differentiate into macrophages in lesions. Wild-type mice with induced endometriosis exhibit increased monocyte recruitment and replenishment of LpM pools from monocytes. In mice where monocyte recruitment is constitutively limited (*Ccr2*^−/−^ or *Ccl2*^−/−^), LpM and SpM pools are significantly reduced, consistent with the majority of LpM in the peritoneal cavity being embryo-derived. In these (monocytopenic) mice, more lesions develop. Mice with ontogenetically reprogrammed peritoneal cavities (embryo-derived LpM depleted using liposomal clodronate followed by a 19-d replenishment window) develop significantly fewer lesions. Collectively, these data suggest that monocyte-derived LpM protect the peritoneal cavity when challenged with ectopic endometrial tissue. We propose a putative model where endometrial macrophages promote lesion growth, while monocyte-derived macrophages (possibly monocyte-derived LpM) protect the peritoneal cavity against establishment of lesions.

Consistent with a significant input of monocytes to LpM in endometriosis, our most striking result was revealed by constitutively limiting monocyte recruitment (using both *Ccr2*^−/−^ and *Ccl2*^−/−^ mice) and subsequently reducing both LpM and SpM replenishment, which resulted in mice with induced endometriosis developing significantly more lesions. When left unmanipulated, female *Ccr2*^−/−^ mice and wild-type controls normally exhibit equivalent numbers of LpM (10). Thus, we suggest that monocyte-derived macrophages act to protect the peritoneal cavity and can limit the establishment of lesions (Fig. 8).

In monocytopenic mice with endometriosis, a reduction in SpM and LpM as well as monocytes was observed. Monocytopenic mice also exhibited significantly more lesions, indicating that monocyte-derived cells are “antiendometriosis,” although to which population of monocyte-derived cells this function can be attributed remains uncertain. As transient antibody-mediated depletion of monocytes during the establishment phase failed to increase the number of lesions but also left SpM and LpM populations intact, it seems likely that either SpM or LpM provide a dominant antiendometriosis effect.

We determined that a significant proportion of LpM (27.4% of lesion resident cells were GFP+ following adoptive transfer of LpM) but not SpM enter endometriosis lesions using adoptive transfers; we suggest that these LpM change phenotype in lesions very rapidly because only a few lesion cells positive for both F4/80 and GATA6 (<1%) were identified using dual staining, whereas LpM in the cavity are almost entirely positive for GATA6 (12). Aside from identifying peritoneal LpM as a source of macrophages in endometriosis lesions, these findings are important because they show that LpM are able to reprogram and survive in ectopic tissue, a topic of significant controversy in the field of tissue-resident macrophage biology (38, 39). Indeed, our results are consistent with the reversible expression of GATA6 by LpM in the absence of sustained retinoic acid receptor signaling (12) and mirror recent findings that pericardial cavity GATA6+ macrophages lose expression of GATA6 following recruitment to areas of ischemic heart disease (40). In the same manner, mature F4/80^hi^ GATA6+ peritoneal LpM are reported to traffic directly across the mesothelium into the liver following sterile injury. Once in the liver, the macrophages undergo local proliferation and up-regulation of markers of alternative activation, such as Arginase 1 (*Arg1*) and *Retnla*. In the absence of peritoneal macrophages, healing was significantly delayed (41). Our data suggest that in endometriosis LpM trafficking to lesions may play a similar role, perceiving the ectopic tissue as a wound and activating repair processes. Interestingly, GATA6-positive macrophages that invade the epicardium from the pericardial space following experimental myocardial infarction were antifibrotic, despite a rapid loss of GATA6 expression (40). Fibrosis is a consistent feature of endometriotic lesions (42). While eutopic endometrium is able to undergo scar-free healing to restore full tissue functionality, when the tissue is translocated to the peritoneal environment fibrotic “repair” occurs to form lesions. The mechanisms responsible for this are yet to be fully resolved but prorepair macrophages have been implicated in the process (43). Thus, it seems unlikely that LpM trafficking into lesions contribute to fibrotic repair; however, the ontogeny of macrophages contributing to fibrogenesis in endometriosis remains to be determined. Interestingly, our data suggest that SpM (which likely include both classical dendritic cells [cDC]1 and cDC2, with the flow cytometry gates used in our study) may have a neutral role in the pathophysiology of endometriosis since they neither increased in number nor appeared to significantly contribute to the lesion-resident population. Moreover, in our final experiment we sought to reprogram the peritoneal cavities of mice such that embryo-derived LpM were replaced by monocyte-derived LpM. In endometriosis mice with reprogrammed cavities significantly fewer lesions developed. Hence, we suggest it is the monocyte-derived LpM that are protective against development of endometriosis.

One limitation of the current study is the interexperiment variation in lesion number and size in wild-type mice. The mouse model of induced endometriosis used in the study relies on spontaneous attachment of the donor endometrial tissue to the peritoneal lining of recipient mice. This avoids the additional inflammation triggered by suturing (used in a number of other models). We carefully control the amount of tissue used to inoculate recipient mice, and any donor mice that do not mount the appropriate decidual response are excluded. One possible cause that may impact the efficacy of endometrial tissue attachment is a discrete difference in the immune status of batches of mice. Future studies will determine ontogenetic and phenotypic differences in peritoneal macrophages recovered from mice with varying degrees of endometrial tissue attachment.

Our data indicate a key role for monocyte recruitment to the peritoneal cavity and ectopic tissue in endometriosis. We readily detected lesion-resident monocytes as well as cells that were double-positive for both Ly6C and F4/80, but whether this indicates that monocytes rapidly differentiate in lesions or that differentiated monocyte-derived macrophages are recruited from the peritoneal cavity remains unclear. We speculate that monocyte-derived cells represent the largest lesion-resident population. However, we cannot currently conclude whether these are monocyte-derived LpM or monocytes recruited directly to the lesion. If the latter, we do not know whether these come via the cavity or through the newly formed vasculature associated with the lesion.

In a previous study, Bacci et al. (24) depleted peritoneal macrophages using liposomal clodronate in a mouse model of endometriosis. Depletion of peritoneal macrophages prior to transfer of endometrial tissue had no significant impact on lesion establishment and growth, implying that embryonic-derived LpM are redundant in lesion development. Continuous depletion during establishment and growth resulted in significantly smaller lesions, allowing the authors to conclude that the dominant role for macrophages in endometriosis is to promote the development of lesions. It may be presumed that this approach has the potential to deplete all monocytes and macrophages, including those in the transferred endometrial tissue. Thus, in light of our findings that donor endometrial macrophages play a significant role in facilitating lesion growth we suggest the findings of Bacci et al. (24) are a consequence of global macrophage depletion. In contrast, our study used depletion strategies that target macrophages from different origins. We demonstrate a protective role for monocyte-derived macrophages (presumably in LpM form). Similarly, further experiments by Bacci et al. demonstrated that i.p. transfer of BM-derived macrophages could enhance or inhibit growth dependent on polarization of macrophages in vitro prior to transfer (24). Further studies will aim to elucidate the exact phenotype and mechanism of monocyte-derived LpM in endometriosis.

Definition of the macrophage populations that reside in diseased tissues is vital for understanding macrophage-driven pathology, particularly for endometriosis where little is known about the lesion macrophage niche. In the future it may be possible to harness the protective properties of monocyte-derived macrophages as a potential therapy for women with endometriosis. We propose a putative model that in endometriosis macrophages derived from the endometrium exhibit “proendometriosis” functions and facilitate growth of endometriotic lesions, whereas monocyte-derived cells, possibly in the form of LpM from the cavity, have an “antiendometriosis” role and are protective against persistence of ectopic tissue and establishment of lesions.

Collectively, we have demonstrated multiple origins for endometriotic lesion-resident macrophages, a key role for monocyte-derived macrophages in protecting the peritoneal cavity when challenged with ectopic endometrial tissue, and a pathological role for endogenous endometrial macrophages. Our findings imply that monocyte recruitment or monocyte-derived macrophages may be defective in women with endometriosis, and this should be explored in more depth in women with the condition. Thus, we have opened up avenues and possibilities for how macrophages can be targeted or harnessed as a therapeutic option in the treatment of endometriosis.

## Materials and Methods

### Animals and Reagents.

Wild-type C57BL/6JOIaHsd female mice were purchased from Harlan (Harlan Sprague Dawley Inc.) at 8 to 12 wk of age. All transgenic lines used in this study were on the C57BL/6 background and were bred and maintained at the University of Edinburgh or the University of Warwick. All animal work was licensed and carried out in accordance with the UK Home Office Animal Experimentation (Scientific Procedures) Act 1986 and the work licensed under PPL 70/8731 (E.G.). Mice had access to food and water ad libitum and were kept at an ambient temperature and humidity of 21 °C and 50%, respectively. Light was provided 12 h a day from 7 AM to 7 PM. Further details are available in *SI Appendix*, *Materials and Methods*.

### Mouse Model of Induced Endometriosis.

Endometriosis was induced in mice using a syngeneic model as previous described (28). The model aims to mirror the process of “retrograde menstruation.” Further details are available in *SI Appendix*, *Materials and Methods*.

### Flow Cytometry.

Endometrium or lesions were dissected, pooled from each mouse, and placed in 2 mL of ice-cold DMEM. Tissues were cut into small pieces using a scalpel and digested with 1 unit of Liberase DL, 1 unit of Liberase TL, and 0.6 mg DNase enzymes. The tissue and enzymes were incubated for 45 min at 37 °C, with vortexing every 5 min. Following digestion samples were filtered through 100-μM filters. Red blood cells were lysed from peritoneal lavages and cells derived from the endometrium or lesions, and approximately 10^6^ cells per sample were blocked with 0.025 mg anti-CD16/32 (clone 93; BioLegend) and then stained with a panel of antibodies shown in *SI Appendix*, Table S1. Brilliant violet stain buffer was included when required. Fluorescence minus one and unstained controls were used to validate gating strategies. Just prior to analysis on the flow cytometer, DAPI and 123count eBeads (Thermo Fisher Scientific) were added to samples. Samples were analyzed using an LSRFortessa with FACSDiva software (BD Biosciences) or FACSMelody with Chorus software and analyzed with FlowJo v.9 software (FlowJo). Analysis was performed on single, live cells determined using forward scatter height vs. area and negativity for live/dead (DAPI or alternative viability dye). For FACS, red blood cell lysis, Fc blocking, and fluorescent staining was performed as previously described and samples were sorted into pure cell populations based on cell surface marker expression using a FACS Fusion (BD Biosciences). For data where peritoneal populations are expressed as cells per microliter, absolute counts were calculated using 123 eBeads and the following equation: absolute count (cells/µL) = (cell count/eBead count) × eBead batch concentration. Final volume for cytofluorimetric analysis was 300 μL. Thus, for example, 400 cells per microliter is equal to 1.2 ×10^5^ cells per cavity.

### Immunofluorescence.

Immunofluorescence was performed as previously described (26, 27, 44). Further details are available in *SI Appendix*, *Materials and Methods*.

### Fiji Analysis.

For cell counting of F4/80, GATA6 dual immunofluorescent stains, four random images at ×63 objective were taken from each lesion and images quantified using Fiji plugin Cell Counter. Total nuclei were counted, as well as cells positive for respective markers. Values were expressed as percent of total DAPI+ cells. The area of hematoxylin/eosin-stained lesions captured using 2.5× magnification was measured in Fiji by setting the scale to a known size (198 pixels = 500 μM) and then drawing around boundary of the lesion (excluding peritoneal and adipose tissue) and using the measure function.

### Definiens Analysis.

Ly6C, F4/80 dual immunofluorescence was automatically quantified using slide scanning and machine learning. Stained tissue sections were imaged on a Zeiss Axioscan.Z1 (Carl Zeiss AG) at 20× using fluorescence filters configured for DAPI, FITC, and Cy3. Whole-slide .czi files were imported into TissueStudio 2.4 (Definiens AG) for automated tissue detection followed by manual correction of regions of interest to delineate endometriosis lesion, peritoneum, hemorrhage, and adipose tissue. TissueStudio’s built-in nuclear segmentation, using the DAPI channel, was applied within these regions to identify cell objects and these objects were then classified as positive or negative for each channel based on intensity thresholds which were used across all samples.

### Statistical Analysis.

Statistical analysis was carried out in GraphPad Prism 7.02. Data were first analyzed for normality using an Anderson Darling normality test. If data were normally distributed, either an ANOVA with a Tukey’s post hoc test (more than two samples) or a *t* test (two samples) was performed. If data were not normally distributed, nonparametric tests were used, either Kruskal–Wallis with a Dunn’s post hoc test (more than two samples) or a Mann–Whitney *U* test (two samples). Statistical significance was reported at *P* < 0.05.

## Supplementary Material

Supplementary File

## Data Availability

All study data are included in the article and/or *SI Appendix*.

## References

[1] J. W. Pollard, Trophic macrophages in development and disease. Nat. Rev. Immunol. 9, 259–270 (2009). 19282852 10.1038/nri2528 PMC3648866

[2] T. A. Wynn, A. Chawla, J. W. Pollard, Macrophage biology in development, homeostasis and disease. Nature 496, 445–455 (2013). 23619691 10.1038/nature12034 PMC3725458

[3] F. Ginhoux, S. Jung, Monocytes and macrophages: Developmental pathways and tissue homeostasis. Nat. Rev. Immunol. 14, 392–404 (2014). 24854589 10.1038/nri3671

[4] F. Ginhoux, J. L. Schultze, P. J. Murray, J. Ochando, S. K. Biswas, New insights into the multidimensional concept of macrophage ontogeny, activation and function. Nat. Immunol. 17, 34–40 (2016). 26681460 10.1038/ni.3324

[5] C. Schulz., A lineage of myeloid cells independent of Myb and hematopoietic stem cells. Science 336, 86–90 (2012). 22442384 10.1126/science.1219179

[6] L. C. Davies, S. J. Jenkins, J. E. Allen, P. R. Taylor, Tissue-resident macrophages. Nat. Immunol. 14, 986–995 (2013). 24048120 10.1038/ni.2705 PMC4045180

[7] C. Ju, F. Tacke, Hepatic macrophages in homeostasis and liver diseases: From pathogenesis to novel therapeutic strategies. Cell. Mol. Immunol. 13, 316–327 (2016). 26908374 10.1038/cmi.2015.104 PMC4856798

[8] Y. Zhu., Tissue-resident macrophages in pancreatic ductal adenocarcinoma originate from embryonic hematopoiesis and promote tumor progression. Immunity 47, 597 (2017). 28930665 10.1016/j.immuni.2017.08.018 PMC5664180

[9] C. C. Bain, S. J. Jenkins, Isolation and identification of murine serous cavity macrophages. Methods Mol. Biol. 1784, 51–67 (2018). 29761387 10.1007/978-1-4939-7837-3_5

[10] C. C. Bain., Long-lived self-renewing bone marrow-derived macrophages displace embryo-derived cells to inhabit adult serous cavities. Nat. Commun. 7, ncomms11852 (2016). 27292029 10.1038/ncomms11852 PMC4910019

[11] C. C. Bain., Rate of replenishment and microenvironment contribute to the sexually dimorphic phenotype and function of peritoneal macrophages. Sci. Immunol. **5**, eabc4466 (2020). 32561560 10.1126/sciimmunol.abc4466 PMC7610697

[12] Y. Okabe, R. Medzhitov, Tissue-specific signals control reversible program of localization and functional polarization of macrophages. Cell 157, 832–844 (2014). 24792964 10.1016/j.cell.2014.04.016 PMC4137874

[13] C. Goudot., Aryl hydrocarbon receptor controls monocyte differentiation into dendritic cells versus macrophages. Immunity 47, 582–596.e6 (2017). 28930664 10.1016/j.immuni.2017.08.016

[14] E. L. Gautier, S. Ivanov, P. Lesnik, G. J. Randolph, Local apoptosis mediates clearance of macrophages from resolving inflammation in mice. Blood 122, 2714–2722 (2013). 23974197 10.1182/blood-2013-01-478206 PMC3795463

[15] X. Zhang., Type I collagen or gelatin stimulates mouse peritoneal macrophages to aggregate and produce pro-inflammatory molecules through upregulated ROS levels. Int. Immunopharmacol. 76, 105845 (2019). 31470266 10.1016/j.intimp.2019.105845

[16] Z. Liu., Fate mapping via ms4a3-expression history traces monocyte-derived cells. Cell 178, 1509–1525.e19 (2019). 31491389 10.1016/j.cell.2019.08.009

[17] S. J. Jenkins., Local macrophage proliferation, rather than recruitment from the blood, is a signature of TH2 inflammation. Science 332, 1284–1288 (2011). 21566158 10.1126/science.1204351 PMC3128495

[18] E. E. Ghosn., Two physically, functionally, and developmentally distinct peritoneal macrophage subsets. Proc. Natl. Acad. Sci. U.S.A. 107, 2568–2573 (2010). 20133793 10.1073/pnas.0915000107 PMC2823920

[19] L. Boring., Impaired monocyte migration and reduced type 1 (Th1) cytokine responses in C-C chemokine receptor 2 knockout mice. J. Clin. Invest. 100, 2552–2561 (1997). 9366570 10.1172/JCI119798 PMC508456

[20] S. J. Jenkins., IL-4 directly signals tissue-resident macrophages to proliferate beyond homeostatic levels controlled by CSF-1. J. Exp. Med. 210, 2477–2491 (2013). 24101381 10.1084/jem.20121999 PMC3804948

[21] A. W. Horne, P. T. K. Saunders, SnapShot: Endometriosis. Cell 179, 1677–1677.e1 (2019). 31951524 10.1016/j.cell.2019.11.033

[22] K. T. Zondervan, C. M. Becker, S. A. Missmer, Endometriosis. N. Engl. J. Med. 382, 1244–1256 (2020). 32212520 10.1056/NEJMra1810764

[23] J. Halme, M. G. Hammond, J. F. Hulka, S. G. Raj, L. M. Talbert, Retrograde menstruation in healthy women and in patients with endometriosis. Obstet. Gynecol. 64, 151–154 (1984). 6234483

[24] M. Bacci., Macrophages are alternatively activated in patients with endometriosis and required for growth and vascularization of lesions in a mouse model of disease. Am. J. Pathol. 175, 547–556 (2009). 19574425 10.2353/ajpath.2009.081011 PMC2716955

[25] A. Capobianco., Proangiogenic Tie2(+) macrophages infiltrate human and murine endometriotic lesions and dictate their growth in a mouse model of the disease. Am. J. Pathol. 179, 2651–2659 (2011). 21924227 10.1016/j.ajpath.2011.07.029 PMC3204092

[26] R. Forster., Macrophage-derived insulin-like growth factor-1 is a key neurotrophic and nerve-sensitizing factor in pain associated with endometriosis. FASEB J. 33, 11210–11222 (2019). 31291762 10.1096/fj.201900797R PMC6766660

[27] E. Greaves., Estradiol is a critical mediator of macrophage-nerve cross talk in peritoneal endometriosis. Am. J. Pathol. 185, 2286–2297 (2015). 26073038 10.1016/j.ajpath.2015.04.012 PMC4530129

[28] E. Greaves., A novel mouse model of endometriosis mimics human phenotype and reveals insights into the inflammatory contribution of shed endometrium. Am. J. Pathol. 184, 1930–1939 (2014). 24910298 10.1016/j.ajpath.2014.03.011 PMC4076466

[29] R. T. Sasmono, E. Williams, Generation and characterization of MacGreen mice, the Cfs1r-EGFP transgenic mice. Methods Mol. Biol. 844, 157–176 (2012). 22262441 10.1007/978-1-61779-527-5_11

[30] F. L. Cousins, P. M. Kirkwood, P. T. Saunders, D. A. Gibson, Evidence for a dynamic role for mononuclear phagocytes during endometrial repair and remodelling. Sci. Rep. 6, 36748 (2016). 27827431 10.1038/srep36748 PMC5101509

[31] N. Zhang., Expression of factor V by resident macrophages boosts host defense in the peritoneal cavity. J. Exp. Med. 216, 1291–1300 (2019). 31048328 10.1084/jem.20182024 PMC6547866

[32] J. Li, K. Chen, L. Zhu, J. W. Pollard, Conditional deletion of the colony stimulating factor-1 receptor (c-fms proto-oncogene) in mice. Genesis 44, 328–335 (2006). 16823860 10.1002/dvg.20219

[33] P. A. Louwe., Recruited macrophages that colonise the post-inflammatory peritoneal niche convert into functionally divergent resident cells. bioRxiv [Preprint] (2020). bioRxiv:10.1101/2020.11.30.404988 (Accessed 2 December 2020).

[34] C. Hogg, A. W. Horne, E. Greaves, Endometriosis-associated macrophages: Origin, phenotype, and function. Front. Endocrinol. (Lausanne) 11, 7 (2020). 32038499 10.3389/fendo.2020.00007 PMC6989423

[35] T. A. Wynn, K. M. Vannella, Macrophages in tissue repair, regeneration, and fibrosis. Immunity 44, 450–462 (2016). 26982353 10.1016/j.immuni.2016.02.015 PMC4794754

[36] T. Lucas., Differential roles of macrophages in diverse phases of skin repair. J. Immunol. 184, 3964–3977 (2010). 20176743 10.4049/jimmunol.0903356

[37] A. S. Care, W. V. Ingman, L. M. Moldenhauer, M. J. Jasper, S. A. Robertson, Ovarian steroid hormone-regulated uterine remodeling occurs independently of macrophages in mice. Biol. Reprod. 91, 60 (2014). 25061095 10.1095/biolreprod.113.116509

[38] Y. Lavin., Tissue-resident macrophage enhancer landscapes are shaped by the local microenvironment. Cell 159, 1312–1326 (2014). 25480296 10.1016/j.cell.2014.11.018 PMC4437213

[39] L. van de Laar., Yolk sac macrophages, fetal liver, and adult monocytes can colonize an empty niche and develop into functional tissue-resident macrophages. Immunity 44, 755–768 (2016). 26992565 10.1016/j.immuni.2016.02.017

[40] J. F. Deniset., Gata6^+^ pericardial cavity macrophages relocate to the injured heart and prevent cardiac fibrosis. Immunity 51, 131–140.e5 (2019). 31315031 10.1016/j.immuni.2019.06.010 PMC7574643

[41] J. Wang, P. Kubes, A reservoir of mature cavity macrophages that can rapidly invade visceral organs to affect tissue repair. Cell 165, 668–678 (2016). 27062926 10.1016/j.cell.2016.03.009

[42] P. Vigano., Time to redefine endometriosis including its pro-fibrotic nature. Hum. Reprod. 33, 347–352 (2018). 29206943 10.1093/humrep/dex354

[43] J. Duan, X. Liu, H. Wang, S. W. Guo, The M2a macrophage subset may be critically involved in the fibrogenesis of endometriosis in mice. Reprod. Biomed. Online 37, 254–268 (2018). 30314882 10.1016/j.rbmo.2018.05.017

[44] E. Greaves., Estrogen receptor (ER) agonists differentially regulate neuroangiogenesis in peritoneal endometriosis via the repellent factor SLIT3. Endocrinology 155, 4015–4026 (2014). 25051436 10.1210/en.2014-1086

