# Supplementary material for: Macrophages inhibit and enhance endometriosis depending on their origin
Source: Proc Natl Acad Sci U S A. 2021 Feb 3;118(6):e2013776118. doi: 10.1073/pnas.2013776118 (PMC8017702; doi:10.1073/pnas.2013776118)
Supplement: Supplementary File [file pnas.2013776118.sapp.pdf]

Supplementary Information Appendix

Figures

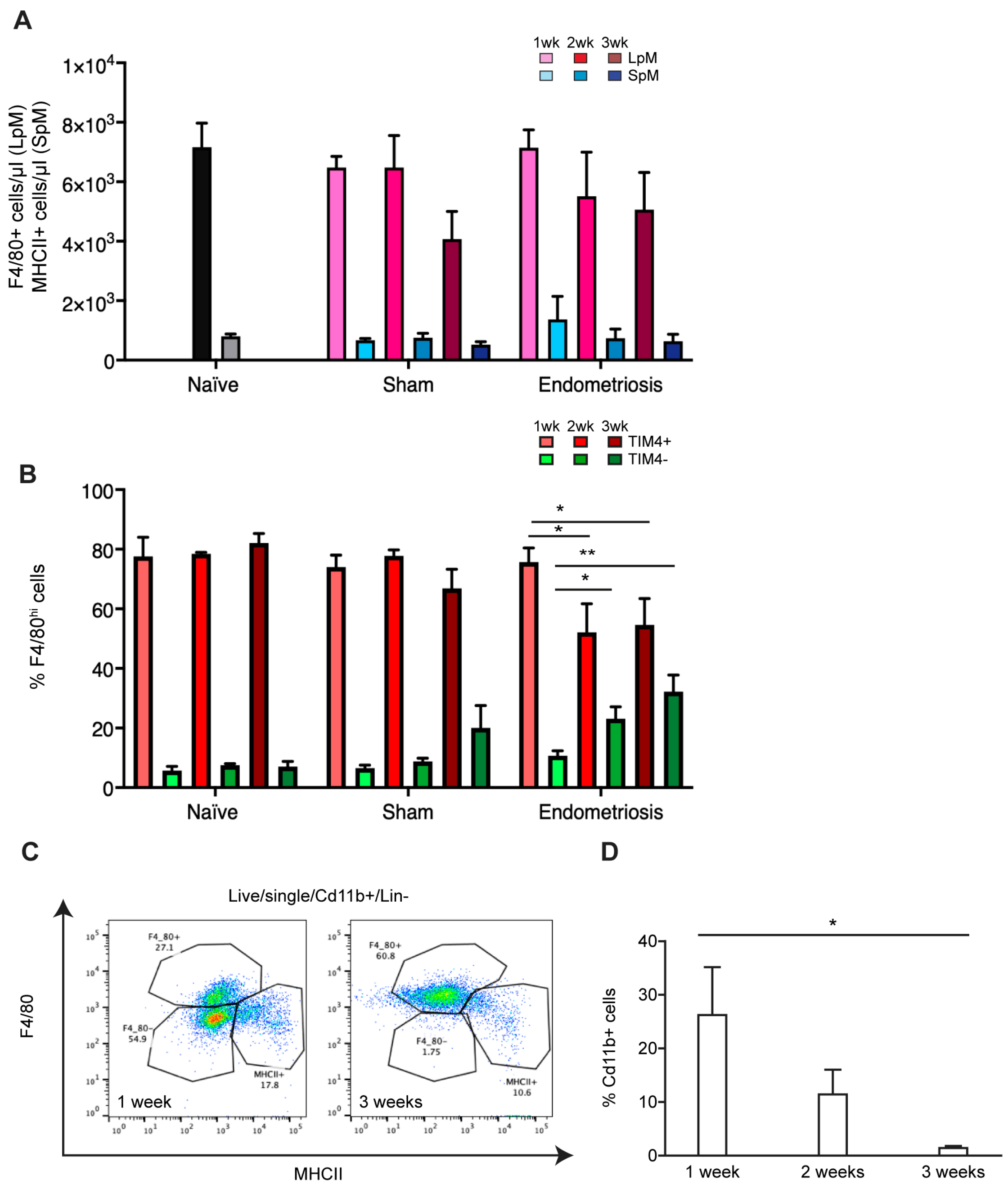

Figure S1: Large and small peritoneal macrophage ratios are not perturbed in a minimally invasive mouse model of endometriosis.

A) Endometriosis was induced in recipient mice that had not had their ovaries removed. LpM and SpM were quantified by flow cytometry at 1 week, 2 weeks, and 3 weeks (n=6-8 per time-point) post tissue injection. Age matched naïve (n=9) and sham (saline instead of tissue, n=3 per time-point) were also included.

B) Quantification of TIM4+ and TIM4- cells in the F4/80^{hi} LpM population present in the peritoneal lavage fluid of mice with endometriosis.

C) LpM and SpM populations in the peritoneal lavage fluid of mice with induced endometriosis 1 and 3 weeks after endometrial tissue injection.

D) Quantification of the F4/80^{lo}, MHCII^{lo} population in peritoneal lavage fluid.

Statistical significance was determined using a two-way ANOVA or Kruskall-Wallis test. *;p<0.05, **;p<0.01.

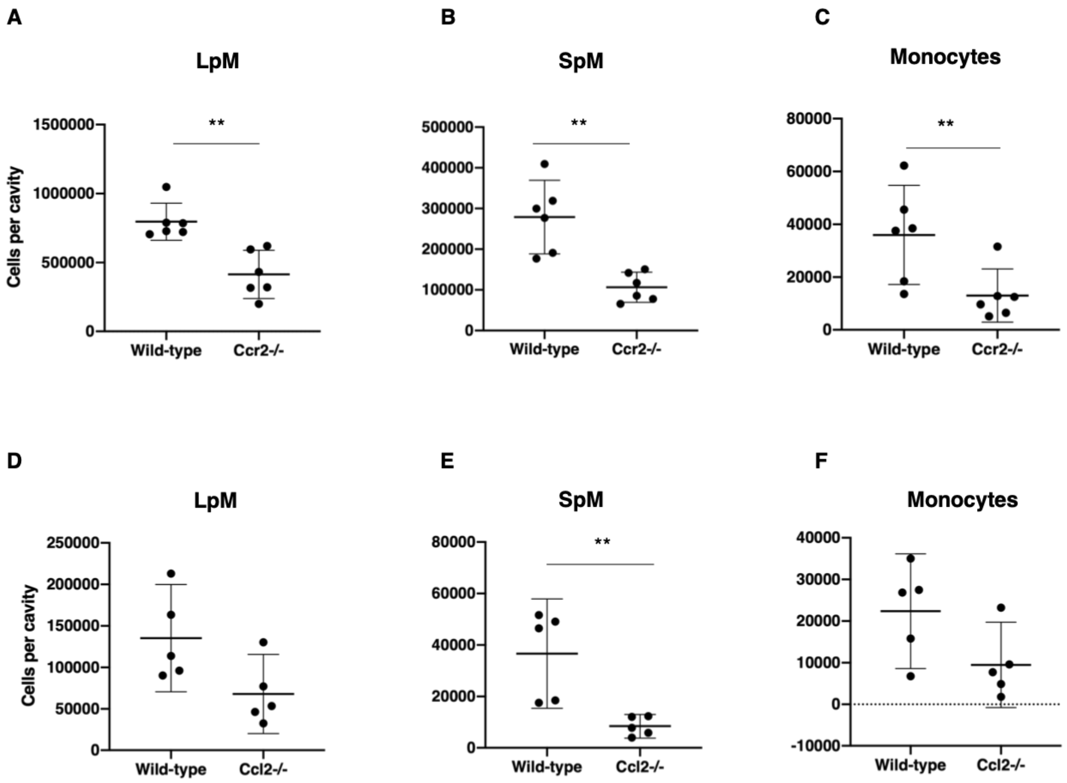

Figure S2: Absolute numbers of LpM, SpM and monocytes in wild-type vs *Ccr2-/-* or *Ccl2-/-* mice with induced endometriosis.

A-C) Quantification of LpM (A), SpM (B), and monocytes (C) per cavity in wild-type vs *Ccr*-/- mice with induced endometriosis.

D-F) Quantification of LpM (D), SpM (E), and monocytes (F) per cavity in wild-type vs *Ccl2*-/- mice with induced endometriosis. Cells per cavity were calculated using count beads during cytofluorimetric analysis.

Data represented are mean with 95% confidence intervals. Statistical significance was determined using a Mann-Whitney test. **;p<0.01.

A

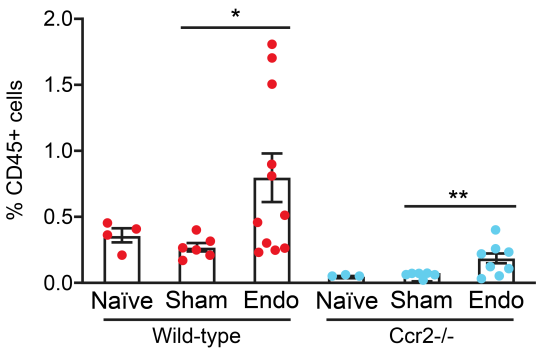

Figure S3: Monocytes can be recruited to the peritoneal cavity of Ccr-/- mice.

A) Quantification of monocytes ($Ly6C^{hi}$ cells) in naïve, sham and mice with induced endometriosis (wild-type vs *Ccr2*-/- mice).

Data are presented as mean ± SEM. Statistical significance was determined using a one-way ANOVA and a Tukey post-hoc test, *;p<0.05, **;p<0.01.

Tables

**Table S1. Flow cytometry antibodies**			
**Antibody**	**Fluorochrome**	**Source**	**Dilution (v/v)**
Tim-4	PE/Cy7	Biolegend	1:200
MHC II	PE/Cy5	Biolegend	1:300
MHCII	AF700	Biolegend	1:400
CD11b	PECF594/ PE/Dazzle	Biolegend	1:300
CD45	PERCP/Cy5.5	Biolegend	1:200
CD3	FITC	Biolegend	1:500
CD19	FITC	Biolegend	1:500
CD335	FITC	Biolegend	1:500
SIGLEC F	FITC	Biolegend	1:500
Ly6G	FITC	Biolegend	1:500
Ly6G	BV650	Biolegend	1:200
Ly6C	PE	Biolegend	1:200
Ly6C	BV711	Biolegend	1:400
F4/80	APC/Cy7	Biolegend	1:500
F4/80	PECy7	Biolegend	1:200
F4/80	PERCP-Cy5.5	Biolegend	1:200
FVS660	APC	Biolegend	1:1000
Dapi			1:10,000

**Table S2**. **Antibodies for immunodetection**						
**Antibody**	**Source**	**Cat number**	**Target cell**	**Species raised**	**Dilution (v/v)**	**Secondary used**
F4/80	eBioscience	14-4801	Macrophage	Rat	1:600	ImmPRESS® HRP-conjugated anti-rat antibody
GATA6	Cell Signalling Technology	5851S	LpM	Rabbit	1:3000	ImmPRESS® HRP-conjugated anti-rabbit antibody
Ly6C	Abcam	Ab15627	Monocytes and monocyte-derived macrophages	Rat	1:100	ImmPRESS® HRP-conjugated anti-rat antibody

Materials and Methods

Animals and reagents. *ROSA26-rtTA:tetO-Cre:Csf1rflox/flox*(33) (colony stimulating factor 1 receptor (Csf1r) conditional knock out) allows deletion of *Csf1r* following treatment with the tetracycline analog doxycycline (2µg/ml in 5% sucrose water; Merck) causing CSF1R expressing macrophage populations to be depleted. *B6.Cg-Tg(Csf1r-EGFP)1Hume/J* (MacGreen) express enhanced green fluorescent protein (EGFP) under control of the *Csf-1r* promoter(1). We used a number of strategies to selectively deplete different monocyte-derived populations in the peritoneal cavity and in lesions; 1) *B6.129S4-Ccr2tm1Ifc/J (C-C chemokine receptor type 2 (Ccr2 -/-)* mice have a homozygous mutation in the *Ccr2* gene(2). They have reduced monocytes, monocyte-derived macrophages and small peritoneal macrophages in the peritoneal cavity in addition to a low number of circulating $Ly6C^{hi}$ monocytes due to an inability for monocytes to extravasate from the bone marrow and from blood vessels. 2) *B6.129S4-Ccl2tm1Rol/J (Chemokine (c-c motif) ligand 2 (Ccl2) -/-)* mice possess a mutation in the *SCYA2* gene encoding the CCL2 ligand. *Ccl2*-/- mice have normal peritoneal macrophage numbers but reduced recruitment of monocytes and monocyte-derived macrophages into the peritoneal cavity under inflammatory conditions(3). 3) The monocyte depleting rat anti-mouse CCR2 mAb (clone MC21) isotype IgG2b(4) was used for monocyte depletion experiments (gifted by Prof M. Mack, University Hospital Regensberg). Mice received a daily intraperitoneal injection of 20µg per mouse of MC21 to prevent infiltration of monocytes into the peritoneal cavity. Isotype-matched rat IgG2b control antibody (MC67) was used as a control. To reprogram the peritoneal cavities of mice such that embryo-derived LpM were replaced with monocyte-derived LpM we administered liposomal clodronate i.p (Encapsula NanoSciences; 0.0625mg/mouse) and allowed replenishment of the niche from monocytes (see below for further details).

Mouse model of induced endometriosis. In brief, donor mice were induced to undergo a 'menses'-like event by removing the ovaries and exposing the mice to a hormonal schedule similar to a truncated menstrual cycle and a stimulus that causes the endometrial stromal cells to undergo decidualization(5). Following P4 withdrawal the endometrial lining begins to shed. 4-6hrs after withdrawal of P4 the 'menses'-like endometrium is collected and injected into ovariectomized mice supplemented with estradiol valerate(5). Lesions are recovered that contain stoma +/- epithelial cells and immune cell influx(6). Unless otherwise stated lesions were collected 2 weeks following tissue injection. For clarity, experiments using different pharmacological / transgenic approaches to deplete monocyte / macrophage

populations and wild-type mice are described: *Experiment 1: Endometrial macrophage incorporation into lesions.* Endometrium from MacGreen donors was injected i.p into wild-type C57BL/6 recipients (n=6 mice). *Experiment 2: Incorporation of peritoneal macrophages into lesions.* LpM (F4/80^{hi}, MHCII^{lo}) or SpM (F4/80^{lo}, MHCII^{hi}) were isolated from MacGreen mice using fluorescent activated cell sorting (FACs) and adoptively transferred into the peritoneal cavity of recipient mice (n=12 per population) at the same time as donor endometrium, both donor and recipient were wild-type C57BL/6. Incorporation was evaluated using immunodetection of GFP. *Experiment 3: Impact of endometrial macrophage depletion on lesion formation.* Macrophages were depleted in donor endometrium by administering doxycycline to *Csf1r*-cKO mice between days 15-19 of the 'menses' protocol (Fig.4A). Macrophage depleted endometrium was injected i.p into wild-type recipients (n=9). For comparison wild-type endometrium was transferred into wild-type recipients (n=10). The data from 2 independent experiments are presented. *Experiment 4: Constitutive depletion of monocytes in the peritoneal cavity (i).* Donor endometrium from wild-type C57BL/6 mice was injected i.p into wild-type (control; n=11) or *Ccr2-/-* recipients (n=13). Data from 3 independent experiments are presented. *Experiment 5: Constitutive depletion of monocytes in the peritoneal cavity (ii).* Wild-type donor endometrium was injected i.p into wildtype (controls; n=6) or *Ccl2-/-* recipients (n=7). Data from 2 independent experiments are presented. *Experiment 6: Transient depletion of monocytes in the peritoneal cavity*. Six hours prior to endometrial transfer recipient C57BL/6 mice received i.p injection of a control IgG (MC67; n=9) or a function blocking CCR2 mAb (MC21; n=10). Syngeneic donor endometrium was then injected i.p into recipients. Mice received daily injections of MC67 or MC21 until day 5 post-tissue injection when mice were culled. Data from 2 independent experiments are presented. *Experiment 7*: *Reprogramming macrophage ontogeny in the peritoneal cavity*. Seven days post ovariectomy C57BL/6 recipient mice received an i.p injection of liposomal clodronate (0.0625mg/mouse, n=10) to deplete peritoneal macrophages. We then waited 19 days to achieve replenishment of the LpM population from monocytes(7). Syngeneic endometrial tissue was injected i.p and lesions recovered on day 5 post tissue injection. Non-depleted control mice (n=10) were ovariectomised at the same time but left unmanipulated until endometriosis tissue transfer. Data from 1 independent experiment is presented. In some experiments we used recipients that had not been ovariectomized (intact). Endometrial tissue was generated and injected in the same way as the standard model. Recipient mice did not receive any hormonal manipulation. In both models, mice were culled 14 days post tissue injection (unless

otherwise stated) and endometriosis lesions and peritoneal lavage were collected. Peritoneal lavage was recovered by injecting 7 ml ice-cold DMEM into the peritoneal cavity followed by gentle massage and recovery. Lesions were either collected into neutral-buffered formalin for paraffin embedding and immunohistochemical analysis or DMEM for flow cytometry analysis.

Immunofluorescence. In brief, sections were antigen retrieved with heat and pressure (buffers pH 6.0 or pH 9.0) or trypsin tablets dissolved in dH2O (for F4/80 antibody; Sigma) and incubated with sections for 20 min at 37°C. Sections were blocked for endogenous peroxidase (6% H_2O_2 in methanol) and nonspecific epitopes (species-specific serum diluted 1:5 in Tris-buffered saline and 5% bovine serum albumin, or blocking serum from species specific ImmPRESS® kit; Vector Laboratories) and incubated with primary antibody (Table 2) at 4°C overnight. Antibody detection was performed using a secondary antibody conjugated to horseradish peroxidase, often from an ImmPRESS® polymer detection kit followed by colour development using a tyramide signal amplification system kit with cyanine (Cy)3 or fluorescein (1:50 dilution; PerkinElmer, Waltham, MA, USA). For detection of the second antigen in dual immunofluorescence, sections were boiled in citrate buffer, and the second primary antibody applied overnight and detected as above. Nuclei were stained with DAPI and sections mounted in Permafluor (Thermo Fisher Scientific). Images were captured on a LSM710 confocal microscope and AxioCam camera (Carl Zeiss). Mouse uterus was used as a positive control tissue, and negative controls had omission of the primary antibody.

References

1. R. T. Sasmono *et al.*, A macrophage colony-stimulating factor receptor-green fluorescent protein transgene is expressed throughout the mononuclear phagocyte system of the mouse. *Blood* **101**, 1155-1163 (2003).
2. L. Boring *et al.*, Impaired monocyte migration and reduced type 1 (Th1) cytokine responses in C-C chemokine receptor 2 knockout mice. *J Clin Invest* **100**, 2552-2561 (1997).
3. B. Lu *et al.*, Abnormalities in monocyte recruitment and cytokine expression in monocyte chemoattractant protein 1-deficient mice. *J Exp Med* **187**, 601-608 (1998).
4. M. Mack *et al.*, Expression and characterization of the chemokine receptors CCR2 and CCR5 in mice. *J Immunol* **166**, 4697-4704 (2001).
5. F. L. Cousins *et al.*, Evidence from a mouse model that epithelial cell migration and mesenchymal-epithelial transition contribute to rapid restoration of uterine tissue integrity during menstruation. *PLoS One* **9**, e86378 (2014).
6. E. Greaves *et al.*, A novel mouse model of endometriosis mimics human phenotype and reveals insights into the inflammatory contribution of shed endometrium. *Am J Pathol* **184**, 1930-1939 (2014).

7. P. A. Louwe *et al.*, Recruited macrophages that colonise the post-inflammatory peritoneal niche convert into functionally divergent resident cells. *bioRxiv* 10.1101/2020.11.30.404988, 2020.2011.2030.404988 (2020).
